# Report of a pertussis outbreak in a low coverage booster vaccination group of otherwise healthy children in Italy

**DOI:** 10.1186/1471-2334-13-541

**Published:** 2013-11-14

**Authors:** Silvio Tafuri, Maria Serena Gallone, Domenico Martinelli, Rosa Prato, Maria Chironna, Cinzia Germinario

**Affiliations:** 1 Department of Biomedical Science and Human Oncology, Aldo Moro University of Bari, Piazza Giulio Cesare 11, 70124 Bari, Italy; 2 Department of Medical and Surgical Science, University of Foggia, Foggia, Italy

**Keywords:** Pertussis, Vaccine failure, Vaccine effectiveness, Booster, Cocoon

## Abstract

**Background:**

The introduction of universal pertussis immunization and the high coverage achieved in most developed countries have largely changed the epidemiology of the disease. Although vaccination rates are high in the first year of life, the rates of booster doses are unsatisfactory and lead to the onset of outbreaks. This report describes an outbreak of pertussis affecting school students already immunized in a town of Puglia (Italy), detected at the end of April 2009.

**Methods:**

Vaccine effectiveness is measured by calculating the incidence rates (attack rates- AR) of disease among vaccinated (ARV) and unvaccinated (ARU) people and determining the percentage reduction in the incidence rate of disease among vaccinated people compared to unvaccinated people.

**Results:**

The index case was a healthy child, female, 9-years-old who attended a local elementary school and developed pertussis on 27 April 2009. The secondary cases were the aunt and the cousin of the index case who developed a cough on 10 May 2009. In the elementary class of the index case, a cluster occurred. The overall AR was 15.8%, in particular 20% in children who did not receive the booster doses at 5–6 years old (ARU) and 14.3% in children receiving the booster (ARV). The VE of booster dose in this setting was 28.5%. Moreover, only the index case developed a persistent cough; the VE against moderate to severe pertussis was 100%. A cluster was detected in the middle school class that the cousin of the index case attended; AR was 44.4% (12/27); ARU was 50% (10/20) and ARV 28.6% (2/7). VE in this setting was 42.8%.

**Conclusions:**

Our results confirm the need to administer booster doses; failure the booster is the principal determinant for the outbreak onset.

## Background

Pertussis is often a mild, unrecognized disease beyond infancy and childhood, and so infected adolescents may serve as the source of infection for unprotected infants in whom pertussis can be severe and sometimes deadly.

A vaccination against pertussis was introduced in the USA in the 1940s. The immunisation program achieved the goal of reducing dramatically the incidence of this disease in infants [1].

In Italy, the vaccination against pertussis has been recommended since 1962. In 1994 the National Health Plan included the advice to offer pertussis vaccination free of charge to all newborns with a 4-dose schedule (3th, 5th, 7th and 13th-19th month) [2]. Despite this National recommendation, a lot of Italian regions didn’t offer the vaccine free of charge and parents had to pay to immunize their children [3].

Since 1995, new acellular vaccines against pertussis have been available and in 1999 the National Immunization Plan provided that a combined vaccine (DTaP) against diphtheria, tetanus and pertussis could be administered during the first years of age using three doses. Booster doses at 5–6 years and 11–12 years of age were also recommended. Each region had to decide autonomously its vaccination strategy (free of charge or in copayment) [4].

In 2002, immunization coverage further improved, after inclusion of pertussis vaccination, in all regions, among the vaccinations to be offered free of charge to all Italian and foreign children [5].

At the national level, vaccination coverage remained low (less than 40%) until early Nineties [3].

Ad hoc studies carried out every five years from 1998 by the National Institute of Health showed a coverage for pertussis of 88% in children born in 1996 and >90% in those born in 2001 and 2006. In 2008 the coverage in adolescents (1992 cohort) was also investigated and the coverage was of 26.7% for 4 doses and 14.1% for five doses [5-7].

The introduction of universal pertussis immunization and the high coverage achieved in most developed countries have largely changed the epidemiology of the disease.

After the introduction of the vaccine and a subsequent substantial decrease in incidence, a resurgence of pertussis has been observed in many countries, particularly in infants and adults.

Before the start of immunization strategies for pertussis, in USA each year 270.000 cases of pertussis and 10.000 deaths were counted. Since the recommendation of the vaccination, the incidence of pertussis dramatically decreased from the 1940’s and in 1976 the minimum number of cases was achieved. Since 1976 the incidence has been increasing and a lot of cases in adolescents and young adults are notified, because of the immunological protection due to the vaccine or natural infection, in absence of boosters (natural or due to the vaccine), wanes over time [8].

In Europe, a resurgence of pertussis has not been consistently documented thus far. Although decreasing trends have been observed in England and Wales, Ireland, Sweden and Spain [9-12] large outbreaks occurred in the Netherlands and Switzerland, and an increasing trend has been observed in Norway [13-19]. In 2007, a cluster of linked cases in adults and adolescents was also described in the United Kingdom [20].

An analysis of current data of 16 European countries in the years 1998–2002 showed that several countries reported the highest incidence of pertussis in children under 1-year-age group. The median age of cases increased from 7 years in 1998 to 11 years in 2002. The incidence in people older than 14 years increased in the study period [21].

In Italy, since the introduction of the pertussis vaccine in 1962, epidemic peaks have regularly occurred every 3–4 years and, despite decreasing incidence and decreasing peak height, no modification in the cycle of the disease was detected up to the 1990’s, with epidemics reported in 1991, 1995 and 1998. Since 1998 no further epidemics have been observed although a slight increase in incidence was reported in 2002 [22].

In 2002 an outbreak occurred in Catania (Sicily, Italy) and 72 people were hospitalized; 30 of them were less than 4-months-old. A cluster in a family was described; the index case was a young adult of 14 years and 10 secondary cases were accounted [23].

Since 1996 high vaccination coverage levels has been maintained in Italy, it was expected that the epidemiological picture of pertussis may substantially change in a few years and become absolutely comparable to that of countries where high coverage levels were reached many years ago.

The following outbreak report describes a cluster of infections affecting school students and some adults in a town of Puglia (Italy).

## Methods

### Study setting

The outbreak occurred in a small community (population: 12.000) in Puglia, Italy, and involved students of local elementary and middle schools and some adults.

Once the outbreak had been detected at the end of April 2009, the investigation involved those cases which had already been reported and those subsequently arising, recorded by notification from the local doctors. The investigation was conducted by the personnel of the Local Health Unit.

A letter was sent to all General Practitioniers and Family Paediatricians, to give information about the outbreak and to invite them to notify all suspected cases; the collection of throat swabs from case-patients for laboratory tests was also required.

The recommended WHO case definition for pertussis was used [24].

Information about age, sex, place of residence, date of occurrence of paroxysmal cough has been collected from children meeting the case definition. Pertussis vaccination history was verified through the immunization registry of the Local Health Unit.

### Laboratory investigation

Throat swabs from case-patients and close contacts were collected and sent to the laboratory of the Hygiene Unit, Azienda Ospedaliera Policlinico Bari, for microbiological tests. Samples were tested for Bordetella pertussis and Bordetella parapertussis with Polimerase Chain Reaction [25].

### Ethical approval

Ethical approval was not required for this study because the project involved data routinely collected for public health purpose. The research was carried out in compliance with the Helsinki Declaration.

Written informed consent for participation in the study was obtained from parent or guardian of children involved.

### Outbreak control measures

Pertussis case patients were excluded from school until five days of recommended antibiotic treatment had been completed. A letter was sent to the parents of elementary and middle school students informing them of the outbreak and recommending a vaccination for students who did not receive booster dose. Antibiotic prophylaxis was recommended for contacts of cases of pertussis.

### Statistical analyses

Vaccination coverage at the start of the outbreak was defined as the proportion of those people who had received a dose of pertussis vaccine during the 5^th^-6^th^ years, as recommended by the National Immunization Plan.

Vaccine effectiveness is measured by calculating the incidence rates (attack rates) of disease among vaccinated and unvaccinated people and determining the percentage reduction in the incidence rate of disease among vaccinated people compared to unvaccinated people. The formula used was:

$$\mathrm{VE}=\frac{\mathrm{ARU}‒\mathrm{ARV}}{\mathrm{ARU}}\times 100$$

where VE = vaccine effectiveness, ARU = attack rate in the unvaccinated population and ARV = attack rate in vaccinated population. 95% CI for VE were also calculated [26].

Data were analyzed using Epi Info 6.0 software.

## Results

**Figure 1:**
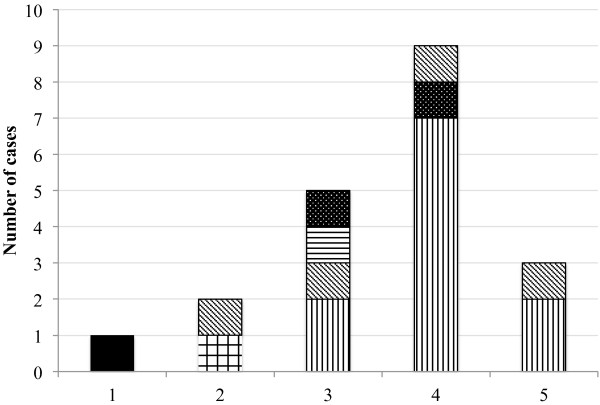
**Number of reported cases of pertussis disease during the outbreak.** Puglia (Italy), April-May 2009. Legend: (■) Index case; (Green square symbol) Adults; (Blue square symbol) Cousin of index cas, student of middle school; (Yellow square symbol) Catechismate of index case; (Purple square symbol) Elementary school; (Red square symbol) Middle school.

**Figure 2:**
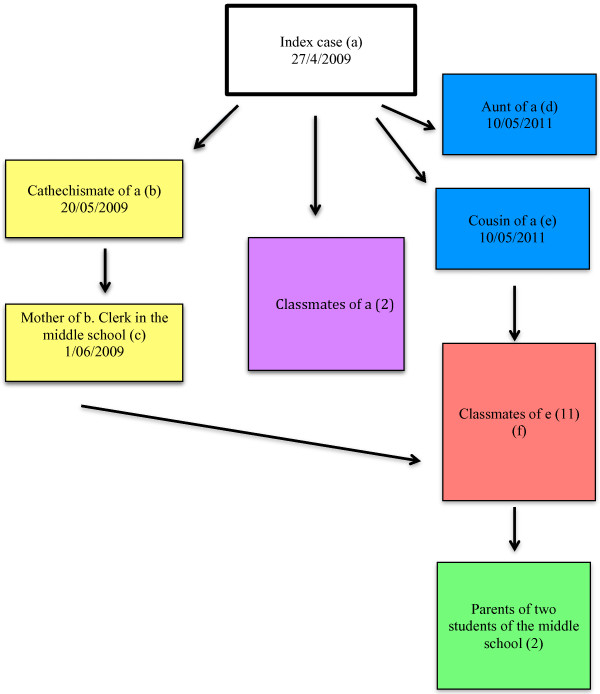
**Diagram of affected contacts.** Cluster of pertussis cases, Puglia (Italy), April-May 2009.

The outbreak started at the end of April 2009 and involved 16 children and four adults (Figure 1, Figure 2).

The index case was a 9-years-old female healthy child, who attended a local elementary school. On 27 April 2009, she developed a cough with post-tussive vomiting, which persisted for eight weeks. She had been immunised against pertussis during the first year with three doses of the vaccine but she did not received a booster dose during the 5^th^-6^th^ year, as recommended by the Regional Immunisation Plan [27]. The source of infection remains unknown (her parents did not report any travel outside her hometown, contact with a pertussis case or having any visitors from abroad in the 21 days before the cough onset).

The secondary cases were the aunt and the cousin of the index case who developed a chronic cough on 10 May 2009. The aunt was 32-years-old not previously immunised for pertussis. The cousin was a 12-years-old boy who attended a local middle school. He received three doses of pertussis vaccine during the first year of his life and another dose in the second year, but he did not receive a booster dose during 5^th^-6^th^ year.

On 20 May 2009, a 9-year-old friend of the index case developed a chronic cough.

The child did not attend the same school of the index case; although she attended the same Sunday school in the local church. She also received three dose of pertussis vaccine the first year of life and another dose in the second year, but she did not receive a booster dose during 5^th^-6^th^ year. On 1 June 2009 her mother, 44-years-old and not previously immunised for pertussis, devoloped a chronic cough. She worked as a clerk in the local middle school.

From 20^th^ May to 1^st^ June, two further cases of pertussis were identified in children attending the same class of the index case. Both received the booster dose at 5–6 years and the symptoms were mild.

Nineteen children attended the class of the index case. All of them received three doses of pertussis vaccination during the first year of life; 14 of them received a booster dose at 5–6 years. The index case did not receive the booster.

In this elementary class, the AR was 15.8% (3/19); in particular it was 20% (1/5) in children who did not receive the booster doses (ARU) and 14.3% (2/14) in children receiving the booster (ARV). The VE of the booster dose in this setting was 28.5% (95% CI = -41.1-96.3). Only the index case developed a persistent cough; the VE against moderate to severe pertussis was 100%.

From 20^th^ May to 4^th^ June 11 cases of pertussis among students attending local middle school were notified; all attended the class of the cousin of the index case. Two of them received the booster dose at 5–6 years and nobody received the booster at 11–12 years. The class was made up of 27 students; 7 received the booster at 5–6 years.

In the middle school AR was 44.4% (12/27); ARU was 50% (10/20) and ARV 28.6% (2/7). VE in this setting was 42.8% (95% CI = -0.36-74.5).

During the outbreak, two cases of pertussis were notified in two adults. They were a father (44-years-old) and a mother (37-years-old) of two secondary cases of the middle school.

Throat swabs were collected from the index case and her close contacts (parents, relatives and classmates), from the adult cases and from 6 students of the middle school. Swabs were tested and all were positive for Bordetella pertussis.

## Discussion

Vaccination coverage is one of the major factors explaining the variation in the incidence of pertussis. Data from the Ministry of Health for Italy showed a high coverage rate in newborns [5-7], but national figures of coverage for booster doses administered at 5^th^-6^th^ year and at 13^th^-14^th^ year are not available and this lack does not allow to predict future trends of pertussis.

Adolescents and adults represent therefore an increasing reservoir of pertussis but may be often unrecognized because they carry the least specific symptoms when affected by the disease.

Our results confirm the need to administer booster doses; failing the booster is the principal determinant for the outbreak onset. In particular, we detected a higher attack rate in the students of the middle school. None received booster dose at 13^th^-14^th^ and only 25% the booster at 5^th^-6^th^ year.

VE was lower than in other studies and this could be partially explained with the use of the clinical case definition in the calculation of VE [28]. In accordance with results by Witt MA et al. published in 2012, the VE did not achieve 50% in elementary school [29]. These data suggest that the current schedule of acellular pertussis vaccine doses is insufficient to prevent outbreaks of pertussis and the possibility of earlier or more numerous booster doses of acellular pertussis vaccine either as part of routine immunization or for outbreak control should be entertained [29].

The observed increase in adolescents and adults might be caused by waning immunity in immunized people. In fact, the immunity induced by immunization is shorter than after the natural disease, and this might explain why pertussis is re-emerging in adolescents and adults in countries with a long history of high vaccination coverage. It is likely that the progressive increase in vaccination coverage and the decrease in frequency of natural boosters will determine more cases in adolescents and adults in Europe [21]. Even if appropriately immunized, while 5 year olds children have approximately 98% protection, that protection wanes to 71% over the next 5 years. So, fundamentally, we don’t have a vaccine that is all that durable over time [30]. Although, some surveys suggest a VE higher than 80% in adolescents who received a booster dose [31]. An ecologic analyse of pertussis notifications in Australia showed that regions administering pertussis vaccine to the entire high school and to subsequent entrant cohorts experienced sustained decreases in pertussis notifications in both adolescents and infants under 6 months of age during 2004–2009 [32].

In our survey, three cases of pertussis in adult people were detected. Some cases in the middle school could be contacts of one of the adults involved in the cluster, who worked in the school as a secretary. Usually clinicians associate pertussis with children and lack of knowledge or appreciation of clinical illness caused by pertussis in adults often renders it unrecognized. Undiagnosed cases contributed to the spread of pertussis to others who are susceptible (e.g., incompletely immunized infants and toddlers) [33]. The General Practitioners must be encouraged in considering the diagnosis of pertussis as part of differential diagnosis of each adult with a prolonged cough. The sensitivity of this case definition is as high as 84-92% [34].

A lot of studies confirmed an high incidence of pertussis among adults; Rendi-Wagner P et al., in 2007, reported that in Austria the mean age of reported pertussis cases increased from 30 years in 2000 to approximately 44 years in 2005 and the hospitalization rate increased by 80% in persons aged 85 years and older [35].

The public health response aimed at ensuring that case and contacts were treated appropriately in limiting infection. Due to the high secondary attack rate associated with unimmunised household contacts, a good family history and prompt notification and testing while providing prophylaxis is crucial in avoiding infection in the most vulnerable population – unimmunised infants [20].

Symptomatic staff were advised not to attend work until five days of recommended antibiotic treatment had been completed.

## Conclusion

Improving the vaccination strategies for the adults is a major objective for Public Health in Europe. Because the outbreaks of pertussis are occurring in Italy, involving adults and infants, the National Society of Public Health (SItI) has recently recommended the use of the trivalent vaccine dTap (instead of the bivalent dT) for adults who have to receive a tetanus booster vaccine every 10 years. SItI recommends that dTap must be used also for the subjects who did not already receive the vaccine against pertussis. Since an increase of the incidence of pertussis in infants (due to close contacts in the family) is expected in the setting with high coverage rates in newborns, SItI recommend to administer a booster dose of dTap for parents and relatives with close contact with the infants before childbirth (*cocoon strategy*) [36,37].

Occupational Health Services, expecially in the school and in Health Trusts, have to implement this recommendation and carry out the promotion of the vaccine against pertussis in adults.

## Abbreviations

ARU: Attack rate in the unvaccinated population; ARV: Attack rate in vaccinated population; VE: Vaccine effectiveness; WHO: World Health Organization.

## Competing interests

The authors declare that they have no competing interests.

## Authors’ contributions

TS and GMS conceived the study, acquired data, carried out the analysis of data and drafted the manuscript. CM performed laboratory investigation. MD and RP revised critically the manuscript. GC revised critically the manuscript and gave the final approval of the version to be published. All authors read and approved the final manuscript.

## Pre-publication history

The pre-publication history for this paper can be accessed here:

http://www.biomedcentral.com/1471-2334/13/541/prepub
